# Editorial: Stem cell research and therapy for liver and kidney diseases

**DOI:** 10.3389/fcell.2025.1665821

**Published:** 2025-07-28

**Authors:** Daojun Lv, Ting Li

**Affiliations:** ^1^ Guangdong Provincial Key Laboratory of Major Obstetric Diseases, Department of Urology, Guangdong Provincial Clinical Research Center for Obstetrics and Gynecology, The Third Affiliated Hospital, Guangzhou Medical University, Guangzhou, China; ^2^ Department of Hepatobiliary Surgery, The Third Affiliated Hospital, Guangzhou Medical University, Guangzhou, China

**Keywords:** stem cell, liver diseases, kidney diseases, therapy, reviews

Liver and kidney diseases remain significant global health challenges, characterized by high morbidity, mortality, and substantial socioeconomic burdens (Mak et al., 2024; Devarbhavi et al., 2023). Specific conditions including hepatocellular carcinoma (HCC), primary sclerosing cholangitis (PSC), and hepatic fibrosis, alongside chronic kidney disease (CKD) and acute kidney injury (AKI) present formidable clinical management difficulties. These challenges arise primarily from limited effective therapeutic strategies and severe shortages of donor organs for transplantation. Stem cell therapy emerges as a promising therapeutic avenue to these limitations by providing regenerative, immunomodulatory, and paracrine therapeutic potential (Francis et al., 2024; Liu et al., 2022). Recent advancements in stem cell technologies, coupled with increasing clinical urgency, further underscore the critical importance and timeliness of developing stem cell-based interventions for hepatic and renal pathologies (Chen et al., 2023; van den Berg et al., 2025). This Research Topic “*Stem cell research and therapy for liver and kidney diseases*” addresses critical knowledge gaps and emphasizes translational progress through a curated selection of one original research study and four review articles.

Currently, stem cell research targeting liver and kidney diseases encompasses multiple cell types and mechanisms. Mesenchymal stem cells (MSCs), particularly bone marrow-derived MSCs (BM-MSCs), demonstrate efficacy against liver cancer through epigenetic modulations and paracrine effects, reducing tumor proliferation and improving survival outcomes *in vitro* and *in vivo* (Johor et al.). Meanwhile, pharmacotherapy for primary sclerosing cholangitis (PSC) faces persistent challenges, where stem cell-based interventions hold promise for addressing inflammation and cholestasis not adequately addressed by current pharmacotherapy (Yang et al.). Liver fibrosis modeling also remains challenging due to the complexity of extracellular matrix accumulation and cellular crosstalk, underscoring the need for advanced, patient-specific, multicellular 3D stem cell models (Ros-Tarraga et al.). In kidney diseases, stem cells provide protective effects *via* immune modulation, paracrine signaling, and direct integration. However, optimizing protocols and ensuring long-term outcomes remain critical translational hurdles (Salybekov et al.). Furthermore, small molecule based chemical approaches offer innovative strategies to enhance hepatocyte transplantation by improving cell quality, differentiation, engraftment, and repopulation rates, essential for clinical translation (Shi et al.).

This Research Topic integrates five insightful contributions addressing pivotal aspects of stem cell therapy for liver and kidney diseases. The review by Yang et al. provides an extensive overview of pharmacological treatments for PSC, highlighting clinical trial progress and uniquely positioning stem cell therapy as a promising adjunct to conventional pharmacotherapy with transformative potential for patient outcomes. Johor et al. demonstrates through rigorous *in vitro* and *in vivo* evaluation that BM-MSC-derived conditioned media synergizes effectively with cisplatin in hepatocellular carcinoma treatment, presenting a viable combined therapeutic strategy. Ros-Tarraga et al. critically evaluates current *in vitro* liver fibrosis models, comparing primary human cells, hepatic cell lines, and stem cell-derived models, emphasizing the advantages and limitations crucial for future model optimization and therapeutic testing. Salybekov et al. outlines advancements in cell therapy for kidney regeneration, detailing preclinical evidence, translational challenges, and potential solutions such as optimized cell sourcing, delivery methods, and dosing strategies, critically shaping the roadmap for successful clinical application. Lastly, Shi et al. reviews novel chemical approaches using small molecules to tackle hepatocyte transplantation barriers, underscoring innovative methods to enhance hepatocyte quality, engraftment, and proliferation, thereby significantly improving therapeutic efficacy and viability.

Collectively, these contributions substantially advance our understanding of the mechanistic potential and translational opportunities for stem cell therapies in liver and kidney diseases. They cohesively underscore critical research breakthroughs, spanning mechanistic insights, innovative therapeutic strategies, and practical clinical solutions. Nevertheless, future research should prioritize addressing persistent challenges, including standardization of cell preparations, deep mechanistic understanding of stem cell interactions with complex disease microenvironments, and robust safety assessments for long-term clinical applications. Encouraging further development in standardized protocols, exploring integrated biomaterials and genetic editing strategies, and executing rigorous GCP-grade clinical trials with long-term follow-up are essential next steps. Ultimately, advancements in these directions promise significant progress towards personalized, effective, and sustainable stem cell therapies, transforming clinical management paradigms for liver and kidney diseases.

## References

[B1] Chen F. Chen N. Xia C. Wang H. Shao L. Zhou C. (2023). Mesenchymal stem cell therapy in kidney diseases: potential and challenges. Cell Transpl. 32, 9636897231164251. 10.1177/09636897231164251 PMC10074620 37013255

[B2] Devarbhavi H. Asrani S. K. Arab J. P. Nartey Y. A. Pose E. Kamath P. S. (2023). Global burden of liver disease: 2023 update. J. Hepatol. 79 (2), 516–537. 10.1016/j.jhep.2023.03.017 36990226

[B3] Francis A. Harhay M. N. Ong A. C. M. Tummalapalli S. L. Ortiz A. Fogo A. B. (2024). Chronic kidney disease and the global public health agenda: an international consensus. Nat. Rev. Nephrol. 20 (7), 473–485. 10.1038/s41581-024-00820-6 38570631

[B4] Liu P. Mao Y. Xie Y. Wei J. Yao J. (2022). Stem cells for treatment of liver fibrosis/cirrhosis: clinical progress and therapeutic potential. Stem Cell Res. Ther. 13 (1), 356. 10.1186/s13287-022-03041-5 35883127 PMC9327386

[B5] Mak L. Y. Liu K. Chirapongsathorn S. Yew K. C. Tamaki N. Rajaram R. B. (2024). Liver diseases and hepatocellular carcinoma in the Asia-Pacific region: burden, trends, challenges and future directions. Nat. Rev. Gastroenterol. Hepatol. 21 (12), 834–851. 10.1038/s41575-024-00967-4 39147893

[B6] van den Berg C. W. Dumas S. J. Little M. H. Rabelink T. J. (2025). Challenges in maturation and integration of kidney organoids for stem cell-based renal replacement therapy. Kidney Int. 107 (2), 262–270. 10.1016/j.kint.2024.10.028 39571903

